# Comparison of Biomarkers in Blood and Saliva in Healthy Adults

**DOI:** 10.1155/2012/246178

**Published:** 2012-04-30

**Authors:** Sarah Williamson, Cindy Munro, Rita Pickler, Mary Jo Grap, R. K. Elswick

**Affiliations:** ^1^Howard Hughes Medical Institute 2010 Summer Scholar, Virginia Commonwealth University, Richmond, VA 23298-0567, USA; ^2^Research and Innovation, University of South Florida College of Nursing, Tampa, FL 33612-4766, USA; ^3^Center for Professional Excellence, Cincinnati Children's Hospital Medical Center, Cincinnati, OH 45229, USA; ^4^Department of Adult Health and Nursing Systems, Virginia Commonwealth University, Richmond, VA 23298-0567, USA

## Abstract

Researchers measure biomarkers as a reflection of patient health status or intervention outcomes. While blood is generally regarded as the best body fluid for evaluation of systemic processes, substitution of saliva samples for blood would be less invasive and more convenient. The concentration of specific biomarkers may differ between blood and saliva. The objective of this study was to compare multiple biomarkers (27 cytokines) in plasma samples, passive drool saliva samples, and filter paper saliva samples in 50 healthy adults. Demographic data and three samples were obtained from each subject: saliva collected on filter paper over 1 minute, saliva collected by passive drool over 30 seconds, and venous blood (3 mL) collected by venipuncture. Cytokines were assayed using Bio-Rad multiplex suspension array technology. Descriptive statistics and pairwise correlations were used for data analysis. The sample was 52% male and 74% white. Mean age was 26 (range = 19–63 years, sd = 9.7). The most consistent and highest correlations were between the passive drool and filter paper saliva samples, although relationships were dependent on the specific biomarker. Correlations were not robust enough to support substitution of one collection method for another. There was little correlation between the plasma and passive drool saliva samples. Caution should be used in substituting saliva for blood, and relationships differ by biomarker.

## 1. Introduction

Many clinical research projects evaluate immune components as biomarkers that reflect patient health status or outcomes of interventions. Innate and adaptive immune components are present in the blood and the saliva and are attractive biomarker targets. Blood is generally regarded as the best body fluid for evaluation of systemic processes. However, collection of blood involves potential risks to subjects, including transient discomfort, bruising, infection at the venipuncture site, and anemia (if large volumes are required or if subjects are vulnerable). Blood collection is also less favored in research involving children as well as in other research subjects for whom venous access is difficult (i.e., elderly or critically ill). While passive drool samples are considered the gold standard for analysis of unstimulated saliva, collection and storage of saliva on filter papers is more convenient. Comparisons of cytokines values in saliva samples obtained by these 2 methods have not been published. This methodologic study compared multiple biomarkers in blood to saliva samples collected in two different ways from human volunteers.

Salivary glands have rich vasculature from which saliva is filtered and processed. Salivary components may originate entirely from the salivary glands or may be derived from the blood by passive diffusion or active transport [1, 2]. In cases where components in saliva are derived from the blood, levels of biochemical and immunological components measured in saliva may reflect blood levels. Substitution of saliva samples for blood in analysis of biomarkers is of considerable interest because collection of saliva is less invasive and does not have any of the risks associated with collection of blood. While there is some information about single biomarkers in saliva (such as cortisol), the correspondence to blood levels varies widely by biomarker, and to date there has been little published regarding how well blood levels of specific cytokines or other biomarkers are represented in saliva, or how saliva collection technique affects recovery of specific biomarkers [3].

 Saliva can be collected and measured as unstimulated whole saliva, unstimulated saliva from specific gland pairs (such as parotid or submandibular-sublingual pairs), or stimulated saliva from specific gland pairs. Unstimulated whole saliva represents the usual, or baseline, saliva present in the oral cavity for the majority of a 24-hour period. Unstimulated whole saliva often correlates to systemic clinical conditions more accurately than stimulated saliva, since materials use to stimulate flow may change salivary composition.

Unstimulated saliva has traditionally been obtained by having the subject seated quietly with his or her head flexed forward and allowing the saliva to passively drip from the mouth to a collection container, or by having the subject gently spit into a collection contain for a specified amount of time. This method of collection is considered the “gold standard” for obtaining many components of saliva [3]. Recently, an alternative method for collection of unstimulated saliva using filter paper placed in the sublingual pocket has been described [4, 5]. Collection of saliva by filter paper has several potential advantages. In contrast to passive drool collection, it does not require active participation of the subject nor upright positioning. Filter paper samples are easy to transport, use less storage space, and can be stored at room temperature.

 Immune components can be measured with commercially available immunoassay kits and have historically been measured using enzyme-linked immunosorbent assays (ELISAs). Such immunoassays are exquisitely sensitive and specific. However, ELISAs generally measure a single biomarker, so sample volume requirements increase dramatically if multiple biomarkers are to be measured. This is particularly problematic for blood collections. New multiplex suspension array technology enables the examination of multiple cytokines and other biomarkers simultaneously from the same small volume sample. Multiplex technologies were originally developed for use with blood samples and more recently have been applied to other fluids such as urine or cerebrospinal fluid; there are few reports of multiplex analysis of saliva.

The specific aim of this research was to compare levels of biomarkers (27 specific cytokines) in 3 sample types (plasma, passive drool saliva, and saliva collected on filter paper) using a single time point, within-subjects correlational design of a convenience sample of healthy volunteer adults. This was undertaken to enable us to evaluate which biomarkers could be reliably determined in less invasive salivary samples, thus reducing the necessity of blood collection in future research, and whether filter paper sampling could be substituted for passive drool collection. 

## 2. Methods

### 2.1. Design

 A single time point, within-subjects correlational design was used.

### 2.2. Setting

 All data collection and analysis were conducted at a certified, core laboratory located in a research intensive university.

### 2.3. Sample

Fifty healthy volunteers were recruited from the medical and academic campuses of a large urban university in the southeastern United States. Inclusion criteria were: age 18 or older and self-identified as healthy. Exclusion criteria were inability to read and understand spoken English (as consent documents and discussion were conducted in English), current pregnancy, or prisoner. Participants were paid a $10 incentive for their time.

### 2.4. Procedures

Approval for the study was obtained from the University's institutional review board. Healthy volunteer subjects were recruited from posted written advertisements placed in public areas on both campuses of the University. Following contact from a potential subject in response to the advertisement, an appointment was made for the individual to come to the laboratory for consent discussion, consent, enrollment, and data collection. Volunteer subjects were asked to avoid eating, drinking, or smoking for one hour prior to appointment time for saliva collection.

Following consent, subjects were asked to complete a short demographic form. Data were collected on additional factors (age, gender, race, ethnicity, and smoking status) in order to provide a comprehensive description of the study sample. Two saliva samples (one filter paper; one passive drool) and a blood sample were collected; collection order was the same for all subjects. For filter paper collection of saliva, Whatman grade 42 filter paper (2.4 cm × 9 cm size) was placed in the sublingual pocket of the participant's mouth for one minute to permit saturation. After the filter paper was removed from the participant's mouth, the furthest extent of the fluid migration on the paper was marked; measurements were used to determine the proportional volume of fluid used to elute salivary components from the filter. Papers were air-dried and then single-packaged in plastic bags to prevent cross-contamination of specimens. For passive drool saliva collection, we instructed the subject to briefly (30-second) refrain from swallowing. We then collected saliva accumulated in the mouth from a single expectoration into a sterile disposable test tube. One vacutainer tube of venous blood (approximately 3 mL of blood in a BD Vacutainer tube preserved with 5.4 mg K2 EDTA) was drawn from the antecubital or other accessible arm vein. Processing of the samples was done following standard laboratory procedures. Blood was centrifuged to obtain plasma, and plasma samples were stored frozen at −70°C until batch assayed. Passive drool saliva samples were stored frozen at −70°C in the collection tube until batch assayed. Filter paper samples were stored at room temperature until assay, and analytes were eluted from the filter paper in preparation for assay, using the method described by Neu and colleagues [5]. When all subjects had been recruited and all samples collected, levels of cytokines in plasma and both saliva samples were determined using a Bio-Plex Suspension Array System (Bio-Rad ) with a commercial 27-plex cytokine detection kit according to the manufacturer's protocol. The 27-plex assay includes human interleukin (IL)-1beta, IL-1ra (receptor agonist), IL-2, IL-4, IL-5, IL6, IL-7, IL-8, IL-9, IL-10, IL-12, IL-13, IL-17, eotaxin, basic fibroblast growth hormone (FGF), growth-colony stimulating factor (G-CSF), granulocyte-macrophage colony-stimulating factor (GM-CSF), interferon (IFN)-gamma, interferon-inducible protein (IP)-10, monocyte chemotactic protein (MCP)-1, macrophage inflammatory proteins (MIPs)-1alpha, MIP-1beta, platelet-derived growth factors (PDGF)-BB, regulated on activation normal T cell expressed and secreted (RANTES), tumor necrosis factor (TNF)-alpha, and vascular endothelial growth factor (VEGF). These 27 cytokines are bundled together in the commercially available cytokine multiplex assay and are commonly used as a panel in assessment of immune function. The cytokines available in this commercially available bundle reflect both pro-and anti-inflammatory immune response. Assays were performed in a core laboratory, where there was extensive experience with measurement of biomarkers from human samples. The Bio-Plex multiplex suspension array assay combines fluorescent flow cytometry and ELISA technology, providing simultaneous quantitation of each of the 27 analytes being assayed in the sample. The manufacturer reports that the assay accurately measures cytokine values in the range of 1–2,500 pg/mL (well within the required limits of detection for this project), is precise (intra-assay CV < 10%, interassay CV < 15%), and shows less than 1% cross-reactivity among cytokines or with other molecules. Output (electronic file results) from the Bio-Plex assays was transferred to an excel Spreadsheet for data analysis.

### 2.5. Data Analysis

Data were analyzed using JMP statistical analysis software. Descriptive statistics were calculated, and scatterplots created for each biomarker. The levels of individual biomarkers were compared using a pairwise correlational analysis. Biomarker data were positively skewed as anticipated, and log transformation was performed to permit approximation of a normal distribution of the data required to calculate correlations.

## 3. Results

 Subjects ranged in age from 18 to 63 years with a mean age of 27.1 years (sd = 9.7). The sample was 52% male. The majority of the subjects were white (72%) and non-Hispanic (90%), 6% were African American, 4% were Asian, 2% were Pacific Islander, and 16% identified themselves as more than one race.

Correlations for cytokines among the plasma, passive drool saliva, and filter paper saliva samples are presented in Table 1. Between passive drool and filter paper saliva samples, statistically significant correlations were found among 16 of the 27 tested cytokines, including IL-1beta, IL-1ra, IL-4, IL-7, IL-8, IL-9, IL-10, IL-12, IL-13, IL-15, G-CSF, IFN-gamma, IP-10, MCP-1, MIP-1beta, and VEGF. Between plasma and passive drool saliva sample, only 3 cytokines were statistically significantly correlated (IL-6, IFN-gamma, and MIP-1beta). No significant correlations were found between cytokine values in plasma samples and filter paper saliva samples.

## 4. Discussion

We undertook this comparison of levels of biomarkers (27 specific cytokines) in 3 sample types (plasma, passive drool saliva, and saliva collected on filter paper) to evaluate which biomarkers in a commercial multiplex panel could be reliably determined in less invasive salivary samples and whether filter paper sampling of saliva could be substituted for passive drool collection. Our data indicate that both the biological fluid and the manner of collection affect measurement of biomarkers. While the greatest associations were between the salivary samples (passive drool and filter paper samples of each biomarker), the relationships varied by biomarker from good correlations to poor correlations. Additionally, although the correlations were statistically significant, no correlation was greater than 0.63, indicating that values obtained from the filter paper were not substitutable for those obtained by passive drool. Passive drool saliva samples were significantly associated with plasma samples for only 3 biomarkers. No significant associations between filter paper saliva and plasma samples were found.

Saliva has been widely analyzed as an indicator of oral health [6–8], and there has been recent interest in use of salivary components as surrogates for systemic biomarkers in blood [9]. This approach has been employed successfully in the case of cortisol [10, 11]. Our data indicate that for some biomarkers, samples collected on filter paper yield different results than passive drool samples. It is likely that some substances bind more tightly to the filter paper and are eluted less efficiently from filter paper; reduced recovery of specific biomarkers from filter paper may account for lack of correlation between passive drool and filter paper samples for several specific biomarkers. The issue of incomplete recovery of selected molecules from matrices used for collection of stimulated saliva (such as cotton wads or cellulose plugs) has been noted in the literature [10, 12, 13].

 Blood still remains the best body fluid for evaluation of many biomarkers reflecting systemic processes and substitution should be used with caution.

Moreover, although salivary levels of cortisol may be reflective of systemic levels, other immune biomarkers in saliva, including IL-6, IL-6sr, and C-reactive protein cytokines, have failed to demonstrate significant correlations to paired samples of plasma [14–16].

 The oral cavity represents a distinct environmental niche, and immune biomarkers are influenced by processes of local immunity. Recently, investigators have identified cytokine levels in saliva associated with gingivitis [6] and periodontitis [7] which reflect local, rather than systemic, immune responses. Therefore, cytokine levels in saliva cannot be generally employed as surrogate markers for systemic immune response.

It may be possible for future studies to use saliva collection as an alternative to blood when measuring specific biomarkers other than cytokines. However, the appropriateness of substitution varies by analyte, and plasma levels of cytokines tested in our panel were generally not well reflected in either type of salivary sample. Further, feasibility of recovery of saliva using the filter paper method and correlation to passive drool salivary values cannot be assumed and should be verified for every biomarker unless published evidence of substitutability is available.

## Figures and Tables

**Table 1:** Correlations of biomarkers among passive drool saliva, filter paper saliva, and plasma samples.

Biomarker	Passive drool versus filter paper	Filter paper versus plasma	Passive drool versus plasma
IL-1*β*	0.63**	0.01	0.12
IL-1RA	0.56**	0.05	0.18
IL-2	0.15	−0.08	0.04
IL-4	0.36*	0.08	−0.18
IL-5	0.17	−0.26	−0.07
IL-6	0.48	0.25	0.31*
IL-7	0.50**	−0.09	0.01
IL-8	0.56**	−0.04	0.17
IL-9	0.53**	0.01	0.23
IL-10	0.52**	0.21	0.28
IL-12	0.62**	0.03	0.26
IL-13	0.37*	0.12	0.12
IL-15	0.43**	−0.03	0.12
IL-17	−0.04	0.05	−0.06
Eotaxin	0.27	0.00	−0.07
Basic FGF	0.01	−0.13	−0.26
G-CSF	0.41**	0.07	0.00
GM-CSF	−0.10	0.13	−0.14
IFN-*γ*	0.33*	0.13	0.34*
IP-10	0.34*	0.25	−0.01
MCP-1	0.44**	0.12	−0.01
MIP-1*α*	0.03	−0.09	−0.10
MIP-1*β*	0.53**	0.29	0.34*
PDGF-BB	0.23	−0.17	−0.22
RANTES	0.24	0.32	0.22
VEGF	0.58**	0.01	0.19
TNF-*α*	0.08	0.19	0.17

^∗^ *P* < 0.05; ^∗∗^ *P* < 0.01.
